# Protura from Hainan Island, China: new species, checklist and distribution

**DOI:** 10.3897/zookeys.879.34404

**Published:** 2019-10-09

**Authors:** Yun Bu, Yan Xiong, Yun-Xia Luan, Wen-Ying Yin

**Affiliations:** 1 Natural History Research Center, Shanghai Natural History Museum, Shanghai Science & Technology Museum, Shanghai, 200041, China Institute of Plant Physiology and Ecology, Chinese Academy of Sciences Shanghai China; 2 Institute of Plant Physiology and Ecology, Chinese Academy of Sciences, Shanghai, 200032, China Shanghai Science & Technology Museum Shanghai China; 3 Shanghai Information Center for Life Sciences, Shanghai Institute of Nutrition and Health, Shanghai Institutes for Biological Sciences, Chinese Academy of Sciences, Shanghai 200031, China Shanghai Institutes for Biological Sciences, Chinese Academy of Sciences Shanghai China; 4 Guangdong Provincial Key Laboratory of Insect Developmental Biology and Applied Technology, Institute of Insect Science and Technology, School of Life Sciences, South China Normal University, Guangzhou, 510631, China South China Normal University Guangzhou China

**Keywords:** distribution, diversity, *
Paracondeellum
*, new species, taxonomy, type specimen

## Abstract

More than 1500 proturan specimens from Hainan Island are systematically studied. An annotated list of all species of Protura from Hainan Island is provided and their geographical distribution is discussed. The genus *Paracondeellum* is reported from Hainan Island for the first time, and *Paracondeellum paradisum***sp. nov.** is described. The type species *Paracondeellum dukouense* (Tang & Yin, 1988) is redescribed based on syntype, and the lectotype and paralectotype are designated. The characters of the genus *Paracondeellum* are redefined, and the two known species are compared in detail. The Protura fauna of Hainan Island is mainly composed of species from the Oriental region, with 91% of the species belonging to the families Berberentulidae and Eosentomidae.

## Introduction

Protura is a group of tiny soil-dwelling arthropods with more than 800 described species (Bu et al. 2012, 2017; Galli et al. 2018). The diagnosis, distribution, and key to 76 known genera and seven families of Protura worldwide were recently given by Galli et al. (2018). So far, there are 214 species belonging to 43 genera recorded in China (Bu et al. 2012, 2017; Qian et al. 2018).

**Figure 1.**
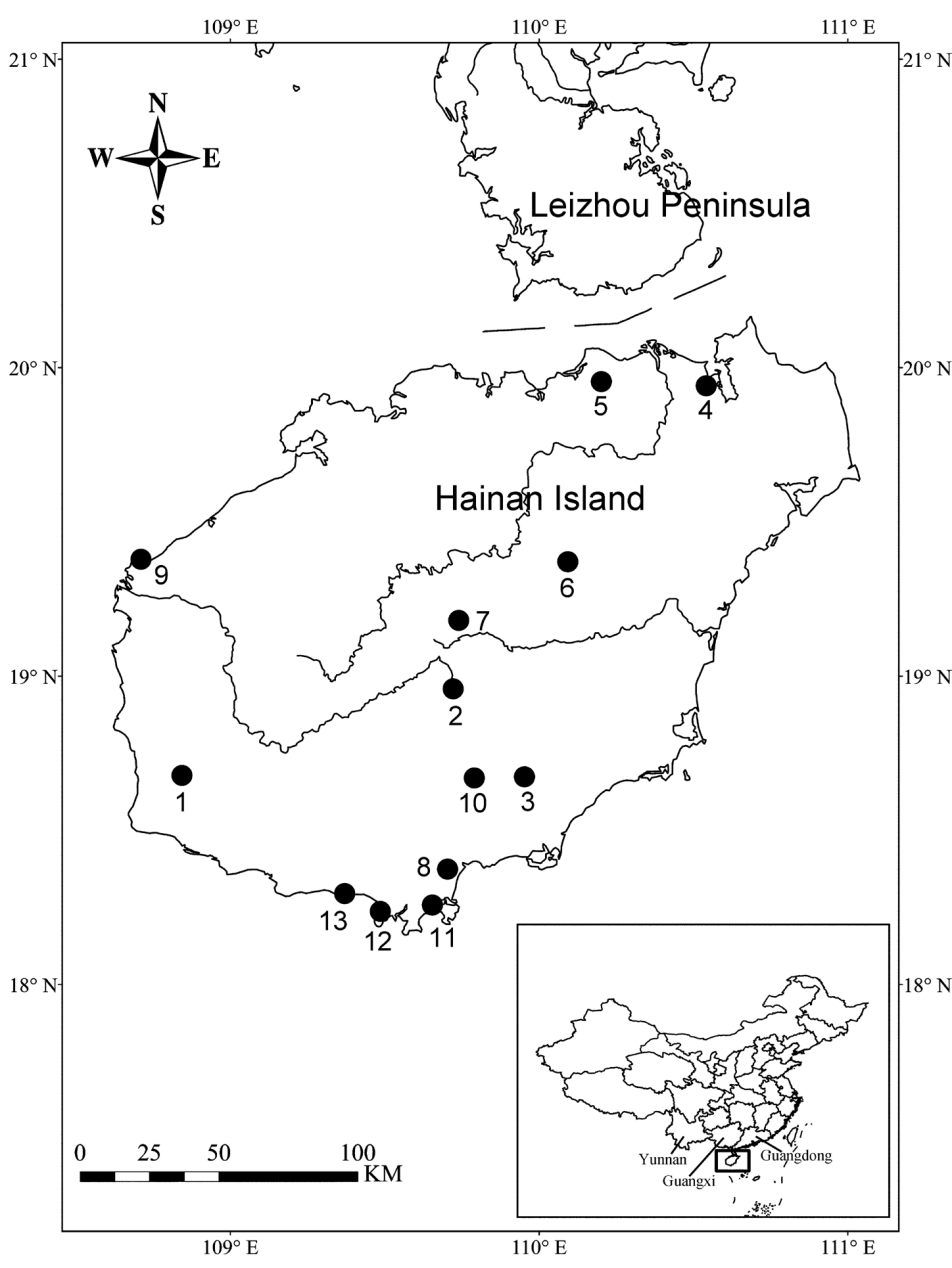
The sampling localities in Hainan Island. Numbers 1–13 indicate the localities listed in Tables 1, 5.

Hainan Island is the second largest island of China and is located off the southernmost point of the mainland (18°10'–20°10'N, 108°37'–111°03'E; Fig. 1). The tropical forest landscape on Hainan Island is one of the hotspots for biodiversity in China, with a high floral diversity and over 6000 species of insects recorded (Huang 2002). In recent years, many rare insects, such as belonging to Zoraptera, have been found on Hainan Island (Yin et al. 2015).

There are several previous publications on the Protura from Hainan Island. The first study reported 14 species of Eosentomidae from Hainan (Yin 1986). Then, eight species of the genus *Kenyentulus* (Berberentulidae) were described (Yin 1987). Later, 24 species of Protura were recorded in Hainan Province with *Fujientomon dicestum* Yin, 1977 and *Pseudanisentomon yongxingense* Yin, 1988 included (Yin 1999, 2002). In 2004, the Protura from Jianfengling Mountain were investigated again. In 2005, *Amphientulus sinensis* Xiong, Xie & Yin, 2005 was described and seven new records and three undetermined species were newly added (Xiong 2005; Xiong et al. 2005). One of these undetermined species was subsequently described as *Anisentomon hainanense* Xiong, Bu & Yin, 2008 (Xiong et al. 2008).

In 2011 and 2017, we investigated the soil fauna of Hainan Island on several occasions and collected many proturan specimens. In the present paper, Protentomidae is recorded for the first time and one new species of genus *Paracondeellum* Yin, Xie & Zhang, 1994 is identified and described. We checked the syntypes of the type species of *Paracondeellum dukouense* (Tang & Yin, 1988), designated a lectotype and paralectotype, and redescribed it in detail. In addition, based on more than 1500 proturans collected in Hainan Island from 1984 to 2017, a checklist is presented and the distribution of Protura on Hainan Island is summarized.

## Materials and methods

Most of specimens were collected between 1984 and 2004, and more recent specimens were collected during the expeditions in 2011 and 2017. All localities sampled so far are listed in Table 1 and shown in Figure 1. All specimens were extracted by means of the Tullgren funnels from soil and humus samples and preserved in 75% ethanol. They were mounted on slides using Hoyer’s solution and dried in an oven at 50 °C.

**Table 1.** The sampling localities of Protura in Hainan Island.

Number	Locality	Coordinates	Altitude (m)	Sampling years
**1**	Ledong County, Jianfengling National Natural Reserve	18°23'– 18°52'N, 108°44'–109°02'E	120–330	1984, 1993, 2003, 2004
**2**	Wuzhishan City, Wuzhishan National Natural Reserve	18°49'–18°59'N, 109°32'–109°43'E	800–1200	1984, 1985, 2004, 2011
**3**	Wuzhishan City, Diaoluoshan National Natural Reserve	18°43'–18°58'N, 109°43'–110°03'E	500–1000	1985, 2004
**4**	Haikou City, Dongzhaigang National Natural Reserve	19°51'–20°01'N, 110°32'–110°37'E	20	2004
**5**	Haikou City, Crater National Geological Park	19°55'N, 110°12'E	223	2003
**6**	Tunchang County, Meiling Mountain	19°22'N, 110°04'E	150–230	2003
**7**	Tunchang County, Limu Mountain	19°17'N, 109°77'E	600–1000	2003
**8**	Baoting County, Ganshenling Provincial Natural Reserve	18°39'N, 109°66'E	500	2003
**9**	Changjiang County, Qizi bay	19°21'N, 108°40'E	15	2011
**10**	Baoting, Qixianling National Forest Park	18°42'N, 109°40'E	150	2017
**11**	Sanya City, Yalong Bay Tropical Paradise Forest Park	18°15'N, 109°38'E	200	2017
**12**	Sanya City, Luhuitou Park	18°13'N, 109°29'E	80	2017
**13**	Sanya City, Sanya bay	18°17'N, 109°22'E	5	2017

Observations were made with a phase contrast microscope (Leica DM 2500). Photos were taken by a digital camera (Leica DMC 4500). Line drawings were made using a drawing tube. All specimens are deposited in the collections of Shanghai Natural History Museum (SNHM) and Shanghai Entomological Museum (SEM), Shanghai, China.

Abbreviations used in the text follow the paper by Bu and Yin (2007). Head setae and pores are named according to Rusek et al. (2012) and Shrubovych (2014). The arrangement of the taxa follows the system proposed by Yin (1999).

## Results

### Taxonomy

#### Family Protentomidae Ewing, 1936

##### Genus Paracondeellum

Taxon classificationAnimaliaProturaProtentomidae

Yin, Xie & Zhang, 1994

AAF9B8EF-C7D8-5C6B-A2AC-539D9FDF5DF9

###### Diagnosis.

Habitus short and robust. Pseudoculi circular without lever. Calyx of maxillary glands globular and smooth. Foretarsal sensilla of the exterior side reduced; interior sensilla *b*’ absent. Abdominal appendages I–II two-segmented each with four setae, III uni-segmented with two setae. Tergites II–VII without or with few anterior setae. Sternites II–III each with three posterior setae. Sternites IV–VII each with nine posterior setae; sternite VIII with four setae in one row. Female squama genitalis short, with pointed acrostyli (Yin 1999; Galli et al. 2018).

###### Distribution.

South China (Sichuan, Yunnan, Hainan).

###### Remarks.

*Paracondeellum* Yin, Xie & Zhang, 1994 was originally separated from the genus *Condeellum* Tuxen, 1963. They have similar shapes of pseudocellus and maxillary gland, and the presence of setae *Pc* on sternites IV–V, but they can be easily separated by the chaetotaxy of tergite I (seta *P5* absent in *Paracondeellum* but present in *Condeellum*) and sternite VIII (four setae in *Paracondeellum* vs six setae in *Condeellum*). In addition, *Paracondeellum* can be distinguished from the genus *Neocondeellum* Tuxen & Yin, 1982 by the shape of pseudocellus (posterior lever absent in *Paracondeellum* but present in *Neocondeellum*) and the chaetotaxy of sternites IV–V (setae *Pc* present in *Paracondeellum* but absent in *Neocondeellum*).

##### Paracondeellum paradisum

Taxon classificationAnimaliaProturaProtentomidae

Bu & Yin sp. nov.

6F18A7F6-9512-58F8-BB0C-AF685065A6D6

http://zoobank.org/A723F8F3-18BF-420F-885E-29EA34F782D7

**Figure 2.**
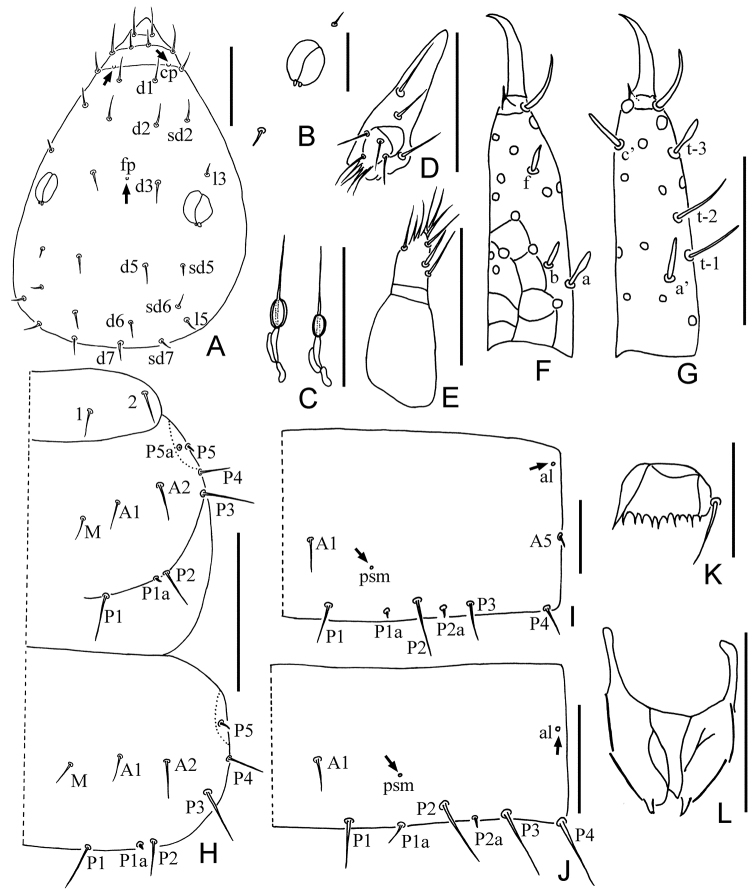
*Paracondeellum paradisum* sp. nov., holotype **A** head, dorsal view (*cp* = clypeal pore, *fp* = frontal pore) **B** pseudoculus **C** canal of maxillary gland **D** labial palpus **E** maxillary palpus **F** foretarsus, exterior view **G** foretarsus, interior view **H** dorsal thorax, right side **I** tergite I, right side (*al* = anterolateral pore, *psm* = posterosubmedial pore) **J** tergite VI, right side **K** comb **L** female quama genitalis. Arrows indicate pores. Scale bars: 10 μm (**B, K**); 20 μm (**A, C–J, L**).

**Figure 3.**
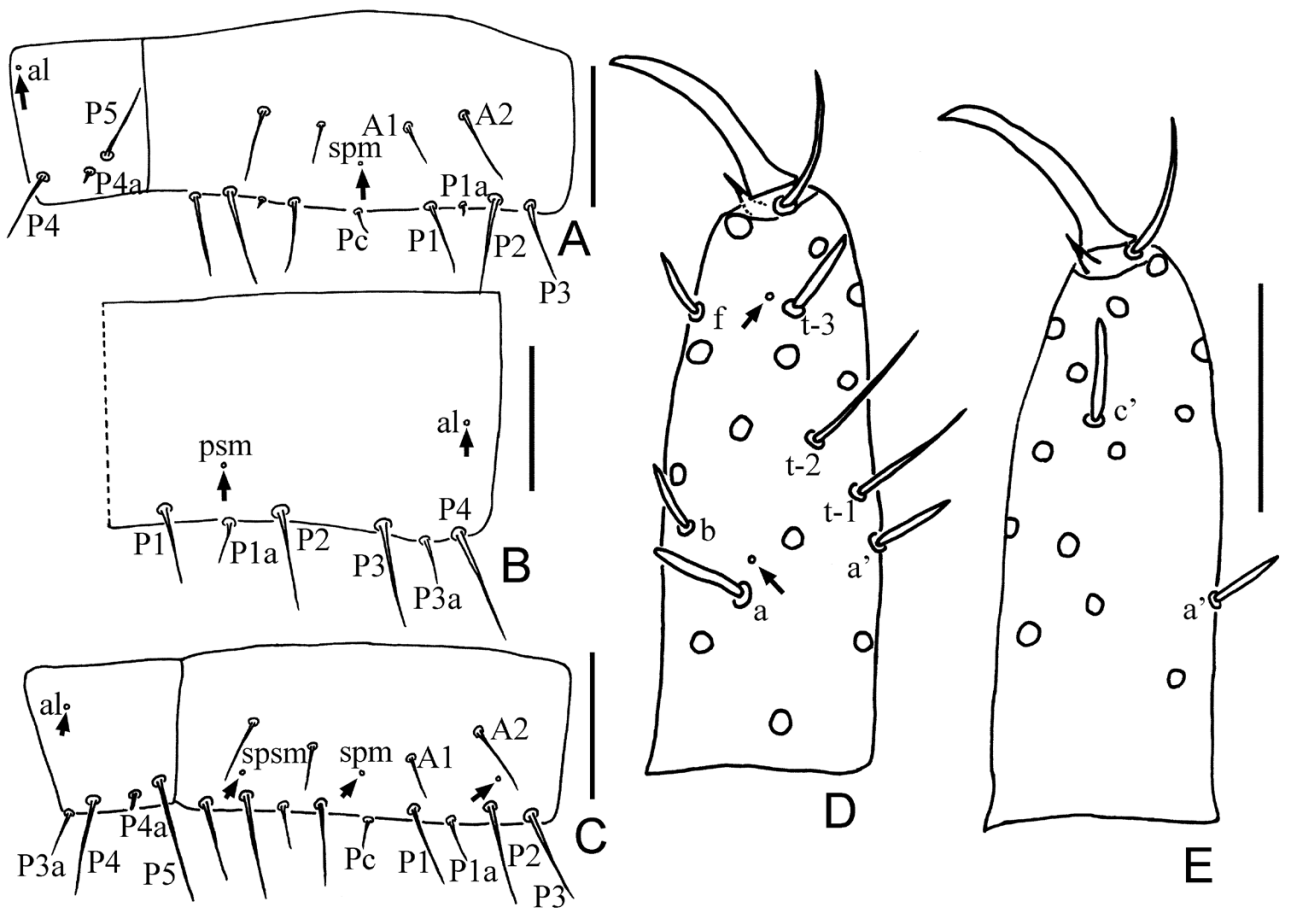
**A–C***Paracondeellum paradisum* sp. nov., holotype **A** sternite VI (spm = sternal posteromedial pore) **B** tergite VII, right side **C** sternite VII (spsm = sternal posterosubmedial pore). **D–E***Paracondeellum dukouense* (Tang & Yin, 1988) holotype **D** foretarsus, exterior view **E** foretarsus, interior view. Arrows indicate pores. Scale bars: 20 μm.

**Figure 4.**
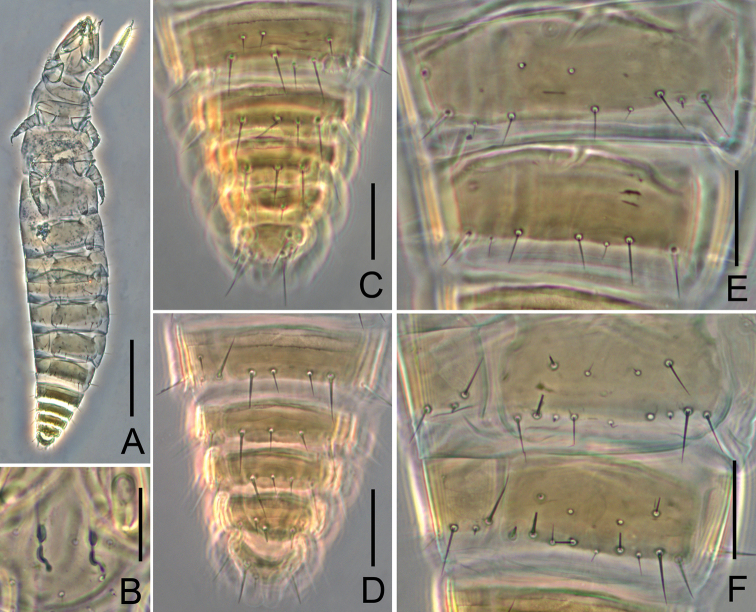
*Paracondeellum paradisum* sp. nov., holotype **A** habitus **B** canal of maxillary gland **C** tergites VIII–XII **D** sternites VIII–XII **E** tergites V–VII **F** sternites VI–VII. Scale bars: 100 μm (**A**); 20 μm (**B–F**).

Figures 2 , 3 , 4 ; Tables 2 , 4

###### Diagnosis.

*Paracondeellum paradisum* sp. nov. is characterized by two pairs of *A*-setae on tergite I, one pair of *A*-setae, and eight pairs of *P*-setae on tergites II–VI, absence of *A*-setae and *P2a* seta on tergite VII, tergites IX and X with 12 and 10 setae, respectively, absence of seta *d4* on dorsal side of head, and female squama genitalis short, with conical acrostylus.

###### Material examined.

Holotype, female (slide no. HN-SY-P2017016) (SNHM), China, Hainan, Sanya City, Yalong Bay Tropical Paradise Forest Park, soil of the tropical rain forest, 200 m elev., 18.25°N, 109.63°E, 22-III-2017, Y. Bu collector. Paratypes, 1 female (slide no. HN-SY-P2017071) (SNHM), same data as holotype.

###### Description.

Holotype: body length 570 μm, yellow-brown, foretarsus darker (Fig. 4A).

*Head*. Elliptic, length 80 μm, width 50 μm (Fig. 2A). Head setae short, rostrum slightly protruded. Setae *d6* and *sd6* present, *d4* and *sd4* absent, *d6* and *d7* length 6 μm and 7 μm respectively. Pores *cp* and *fp* present. Pseudoculus oval, without lever, length 8 μm, width 6.5 μm. PR = 10 (Fig. 2B). Canal of maxillary gland short, with globular calyx and short sausage-like posterior dilation. CF = 10 (Figs 2C, 4B). Labial palpus well developed, with four setae and apical tuft, without basal sensillum (Fig. 2D). Maxillary palpus with two subequal seta-like sensilla (Fig. 2E).

*Foretarsus*. Length 31 μm, claw length 9 μm, TR = 3.4; empodium length 2 μm, EU = 0.22. Dorsal sensilla *t-1* and *t-2* slender and long, BS = 0.63; *t-3* short and spatulate, not reaching base of claw (Fig. 2G). Exterior side with only sensilla *a*, *b* and *f* present; *a* spatulate, *b* and *f* short (Fig. 2F). Interior sensilla a’ and *c*’ short sward-like, *b*’ absent. Relative length of sensilla: *t-2 > t-1 > c' > t3 > a > a' > (b = f)* (Fig. 2F, G). Length of middle tarsus 15 μm; claw length 10 μm. Length of hind tarsus 17 μm; claw length 12 μm.

*Thorax*. Thoracic chaetotaxy given in Table 2. Setae *1* and *2* on pronotum subequal in length, 6 μm and 7 μm respectively (Fig. 2H); mesonotum with seven pairs of posterior setae, *P5a* minute; metanotum with six pairs of posterior setae, *P5a* absent; setae *P1*, *P1a*, *P2* on mesonotum 6 μm, 1 μm, 7 μm, respectively; *P1a* on meso- and metanotum short, pin-shaped (Fig. 2H). Prosternum without seta *A2*. All setae on thoracic sternites of normal shape. Pores on thorax not observed.

**Table 2.** Adult chaetotaxy of *Paracondeellum paradisum* sp. nov.

Segment		Dorsal		Ventral	
		Formula	Setae	Formula	Setae
Th.	I	4	*1*, *2*	(2+2)/6	*A1*, *M*
					*P1*, *2*, *3*
	II	6/14	*A2*, *4*, *M*	(4+2)/4	*A1*, *2*, *M*
			*P1*, *1a*, *2*, *3*, *4*, *5*, *5a*		*P1*, *2*
	III	6/12	*A2*, *4*, *M*	(6+2)/4	*A1*, *2*, *3*, *M*
			*P1*, *1a*, *2*, *3*, *4*, *5*		*P1*, *2*
Abd.	I	4/12	*A1*, *5*	4/2	*A1*, *2*
			*P1*, *1a*, *2*, *2a*, *3*, *4*		*P1*
	II–III	2/16	*A1*	4/3	*A1*, *2*
			*P1*, *1a*, *2*, *2a*, *3*, *4*, *4a*, *5*		*Pc*, 2
	IV–VI	2/16	*A1*	4/9	*A1*, *2*
			*P1*, *1a*, *2*, *2a*, *3*, *4*, *4a*, *5*		*Pc*, *1*, *1a*, *2*, *3*
	VII	0/16		4/9	*A1*, *2*
			*P1*, *1a*, *2*, *3*, *3a*, *4*, *4a*, *5*		*Pc*, *1*, *1a*, *2*, *3*
	VIII	4/14	*A1*, *3*		
			*P1*, *1a*, *2*, *2a*, *3*, *3a*, *4*	4	*1*, *2*
	IX	12	*1*, *1a*, *2*, *2a*, *3*, *4*	4	*1*, *2*
	X	10	*1*, *2*, *2a*, *3*, *4*	4	*1*, *2*
	XI	6		6	*1*, *2*, *3*
	XII	9		6	

*Abdomen*. Abdominal chaetotaxy given in Table 2. Tergite I with two pairs of anterior setae (*A1*, *A5*) and six pairs of posterior setae, *A5* short, sensillum-shaped (Fig. 2I). Tergites II–VI with one pair of anterior (*A1*) and eight pairs of posterior setae, *P2a* present and *P3a* absent (Figs 2J, 3A, 4E, F). Tergite VII without anterior setae and with eight posterior setae, *P2a* absent and *P3a* present (Figs 3B, C, 4E, F). Accessory setae *P1a* on tergites I–V short pin-shaped (4 μm), on tergites VI–VII normal (5 μm). Accessory setae *P2a* and *P4a* always pin-shaped, 2 μm in length. *P3a* on tergite VII of normal shape and 5 μm long (Fig. 4E, F). Tergite VIII with two pairs of anterior setae (*A1*, *A3*) (Fig. 4C). Posterior central seta *Pc* present on sternites IV–VII, sensillum shaped, 4–5 μm long (Figs 3A, C, 4F). *P1a* on sternites IV–VI short, pin-shaped (Fig. 3A), on sternite VII setiform (Fig. 3C).

Tergites I–VII with pores *psm* and *al* (Fig. 2I, J), VIII with pores *psm* only, IX–XI without pores, XII single median pore. Sternites I–VI each with single posteromedial pore *spm* (Figs 3A, 4F), VII with three posteromedial pores (Figs 3C, 4F), VIII with single posteromedial pore (Fig. 4D), IX–XI without pores, XII with one pair of anterolateral *sal* pores.

Abdominal appendages typical of the genus. Subapical setae and apical setae on appendage III 11 μm and 5 μm long respectively. Striate band on abdominal segment VIII reduced to a single serrate line (Fig. 4D). Comb on abdomen VIII rectangular, with 10 teeth, 10 μm wide (Fig. 2K). Female squama genitalis short, with conical acrostylus (Fig. 2L).

###### Etymology.

Latin “paradisum”, after “Paradise Forest Park” where type specimens were collected.

###### Distribution.

China (Hainan)

###### Remarks.

The genus *Paracondeellum* Yin, Xie & Zhang, 1994 is endemic to China and was previously known by a single species, *P. dukouense*, from Sichuan and Yunnan provinces. *Paracondeellum paradisum* sp. nov. differs from *P. dukouense* in the shape of foretarsal sensilla, pseudoculus, and female squama genitalis, and in the body chaetotaxy. A comparison of the morphology of these two species is given in Table 4.

##### Paracondeellum dukouense

Taxon classificationAnimaliaProturaProtentomidae

(Tang & Yin, 1988)

F8ED0826-3C27-5520-AC8B-6EA3D4CA092B

**Figure 5.**
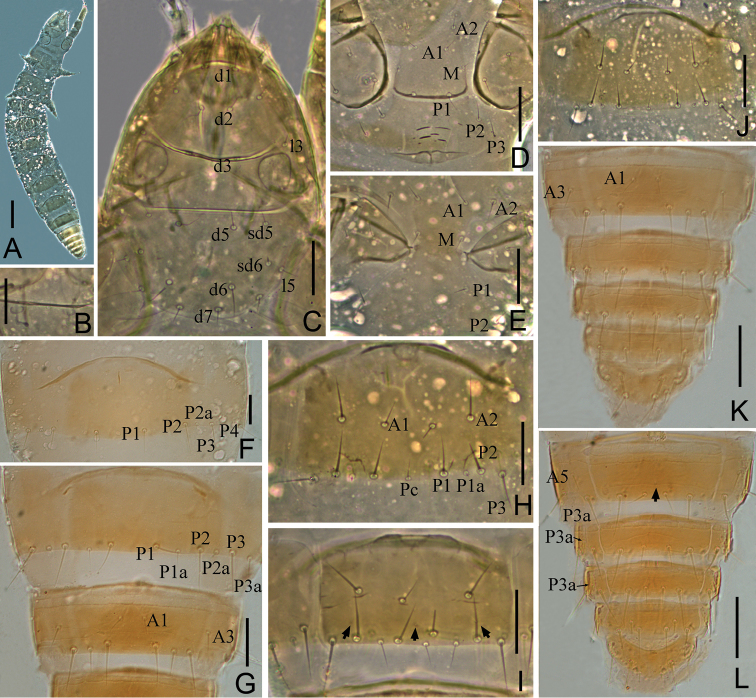
*Paracondeellum dukouense* (Tang & Yin, 1988), holotype **A** habitus **B** canal of maxillary gland **C** head, dorsal view **D** prosternum **E** mesosternum **F** tergite VI **G** tergites VII–VIII **H** sternite VI **I** sternite VII **J** sternite V **K** tergites VIII–XII **L** sternites VIII–XII. Arrows indicate pores. Scale bars: 20 μm.

Figures 3 , 5 ; Tables 3 , 4

###### Diagnosis.

*Paracondeellum dukouense* (Tang & Yin, 1988) is characterized by the one pair of *A*-setae on tergite I, absence of *A*-setae and *P1a* seta on tergites II–VI, absence of *A*-setae and nine pairs of *P*-setae (*P2a* present) on tergite VII, tergites IX and X with 14 and 12 setae respectively, absence of seta *d4* on head, and female squama genitalis with pointed acrostylus.

###### Material examined.

Lectotype, female (slide no. 1), paralectotype, female (slide no. 2) (SEM), China, Sichuan, Dukou City (currently, Panzhihua City), Jinjiang County, soil under grass, 1155 m elev., 26.55N, 101.85E, 26-IX-1985, B.W. Tang and G.T. Jin collectors. We designated as the lectotype the female on slide no. 1 and the other female on slide no. 2 as the paralectotype.

###### Redescription.

Body length of holotype 880 μm and paratype 720 μm; yellow-brown, with foretarsus darker (Fig. 5A).

*Head*. Elliptic, length 93–100 μm, width 70 μm. Dorsal setae longer than subdorsal and lateral ones, rostrum slightly protruded (Fig. 5C). Setae *d6* and *sd6* present, *sd6* sensillum-shaped; *d4* and *sd4* absent; *d6* and *d7* 11 μm and 6 μm long, respectively (Fig. 5C). Pores *cp* and *fp* present. Pseudoculus round, without lever, length 13 μm, width 11 μm. PR = 7.2–7.7 (Fig. 5C). Canal of maxillary gland short, with globular calyx and sausage-like posterior dilation. CF = 13.3–14.3 (Fig. 5B). Labial palpus well developed, with four setae and apical tuft, without basal sensillum. Maxillary palpus with two subequal sensilla.

*Foretarsus*. Length 46–50 μm, claw length 15–17 μm, TR=2.9–3.1; empodium length 4–5 μm, EU=0.24–0.33. Dorsal sensilla *t-1* and *t-2* slender and long, BS=0.66; *t-3* short sward-like, nearly reaching base of claw (Fig. 3D). Exterior slide with only sensilla *a*, *b* and *f* present; *a* spatulate, *b* and *f* short sward-like (Fig. 3D). Interior sensilla *a*’ and *c*’ short sward-like, *b*’ absent (Fig. 3E). Relative length of sensilla: *t-2 > t-1 > c' > a > t3 > a' > (b = f)* (Fig. 3D, E). Length of middle tarsus 20 μm; claw length 12 μm. Length of hind tarsus 23 μm; claw length 15 μm.

*Thorax*. Thoracic chaetotaxy given in Table 3. Setae *1* and *2* on pronotum subequal in length, 10 μm long; mesonotum with seven pairs of posterior setae, *P5a* minute; metanotum with six pairs of posterior setae, *P5a* absent; setae *P1*, *P1a*, *P2* on mesonotum 10 μm, 1.5 μm, 14 μm respectively; *P1a* on meso- and metanotum short, pin-shaped. Prosternum with anterior seta *A2* (Fig. 5D), meso- and metasternum each with four posterior setae (Fig. 5E), metasternum with six anterior setae. All setae on sterna normal. Pores on thorax not detectable.

**Table 3.** Adult chaetotaxy of *Paracondeellum dukouense* (Tang & Yin, 1988).

Segment		Dorsal		Ventral	
		Formula	Setae	Formula	Setae
Th.	I	4	*1*, *2*	(4+2)/6	*A1*, *2*, *M*
					*P1*, *2*, *3*
	II	6/14	*A2*, *4*, *M*	(4+2)/4	*A1*, *2*, *M*
			*P1*, *1a*, *2*, *3*, *4*, *5*, *5a*		*P1*, *2*
	III	6/12	*A2*, *4*, *M*	(6+2)/4	*A1*, *2*, *3*, *M*
			*P1*, *1a*, *2*, *3*, *4*, *5*		*P1*, *2*
Abd.	I	2/12	*A5*	4/2	*A1*, *2*
			*P1*, *1a*, *2*, *2a*, *3*, *4*		*P1*
	II–III	0/14		4/3	*A1*, *2*
			*P1*, *2*, *2a*, *3*, *4*, *4a*, *5*		*Pc*, *2*
	IV–VI	0/14		4/9	*A1*, *2*
			*P1*, *2*, *2a*, *3*, *4*, *4a*, *5*		*Pc*, *1*, *1a*, *2*, *3*
	VII	0/18		4/9	*A1*, *2*
			*P1*, *1a*, *2*, *2a*, *3*, *3a*, *4*, *4a*, *5*		*Pc*, *1*, *1a*, *2*, *3*
	VIII	6/14	*A1*, *3*, *5*		
			*P1*, *1a*, *2*, *2a*, *3*, *3a*, *4*	4	*1*, *2*
	IX	14	*1*, *1a*, *2*, *2a*, *3*, *3a*, *4*	4	*1*, *2*
	X	12	*1*, *2*, *2a*, *3*, *3a*, *4*	4	*1*, *2*
	XI	6		6	*1*, *2, 3*
	XII	9		6	

*Abdomen*. Abdominal chaetotaxy given in Table 3. Tergite I with one pair of anterior setae (*A5*) and six pairs of posterior setae, *A5* short, sensillum-shaped. Tergites II–VI without anterior setae and seven pairs of posterior setae, *P2a* present, *P1a* and *P3a* absent (Fig. 5F). Tergite VII without anterior setae and with nine pairs of posterior setae, both *P2a* and *P3a* present (Fig. 5G). Accessory setae *P2a* and *P4a* on tergites II–VII short, sensillum-shaped, 4 μm in length, *P1a* and *P3a* on tergites VII normal, 9–10 μm in length (Fig. 5F, G). Tergite VIII with three pairs of anterior setae (*A1*, *A3*, *A5*) and seven pairs of posterior setae, *P3a* short (5 μm) (Fig. 5G, K, L). Posterior central seta *Pc* present on sternites IV–VII slender, 8–9 μm long (Fig. 5H–J). *P1a* on sternites IV–VI short pin-shaped, 2 μm long (Fig. 5H, J), on sternite VII as normal seta, 9 μm long (Fig. 5I). Sternites IX and X with short *P3a* seta (Fig. 5L), which had been omitted in original description.

Tergites I–VII with pores *psm* and *al*, VIII with pores *psm* only, IX–XI without pores, XII with single median pore. Pores on sternites I–VI not observed due to the opacity of the old specimens (Fig. 5H, J); three posteromedial pores observed on sternite VII (Fig. 5I), VIII with posteromedial pore (Fig. 5L), IX–XI without pores, XII with one pair of *sal* pore.

Abdominal appendages typical of the genus. Subapical setae and apical setae on appendage III 12–13 μm and 6–8 μm long, respectively. Striate band on abdominal segment VIII reduced to a single serrate line (Fig. 5G, K, L). Comb on abdomen VIII rectangular, with 10 teeth, 12–13 μm wide (Fig. 5K). Female squama genitalis short, with pointed acrostylus.

###### Etymology.

Named for Dukou City (now Panzhihua City, Sichuan Province) where type specimens were collected.

###### Distribution.

China (Sichuan, Yunnan).

###### Remarks.

*Paracondeellum dukouense* was originally described based on two syntypes (Tang and Yin 1988). In the original description (Tang and Yin 1988) and in the monograph of Yin (1999), most important characters such as foretarsal sensilla, pseudoculus, maxillary gland, as well as body chaetotaxy were briefly described and illustrated. After careful study of type specimens under a modern phase contrast microscope with higher resolution, we find that sensillum *c*’ is present on the foretarsus and that some of the setae on the body were previously ignored due to the lower resolution of the microscope used. We correct here these mistakes in the original description and supplement the description of head chaetotaxy, the porotaxy, and the shapes of setae on the body. Table 4 compares *P. dukouense* with the new species.

**Table 4.** Comparison between *Paracondeellum paradisum* sp. nov. and *P. dukouense* (Tang & Yin, 1988).

	*Paracondeellum paradisum* sp. nov.	*P. dukouense*
body length (μm)	570	720–880
pseudoculus (μm)	8	13
foretarsus (μm)	31	46–50
sensilla *b* and *f*	short, rod-like	longer, sward-like
sensillum *t-3*	short and spatulate	longer, sward-like
*A*-setae on tergite I	4 (*A1*, *A5*)	2 (*A5*)
*A*-setae on tergites II–VI	2 (*A1*)	0
*P*-setae on tergites II–VI	16 (*P1a* present)	14 (*P1a* absent)
*P*-setae on tergite VII	16 (*P2a* absent)	18 (*P2a* present)
*A*-setae on tergite VIII	4 (*A1*, *A3*)	6 (*A1*, *A3*, *A5*)
setae on tergite IX	12 (*P3a* absent)	14 (*P3a* present)
setae on tergite X	10 (*P3a* absent)	12 (*P3a* present)
*A*-setae on prosternum	2 (*A2* absent)	4 (*A2* present)

#### List of species from Hainan Island

##### Family Protentomidae Ewing, 1936

###### Paracondeellum paradisum sp. nov.

Taxon classificationAnimaliaProturaProtentomidae

14AEAD6E-C784-589E-984F-F347E2926AE1

####### Description.

The description is given above.

##### Family Berberentulidae Yin, 1983

###### Baculentulus tienmushanensis

Taxon classificationAnimaliaProturaBerberentulidae

(Yin, 1963)

BC4E1EF5-F991-5ECB-BC70-7894EC060EDC

####### Material examined.

1 male, 1 mj, locality 3, 19-I-1985, coll. G. T. Jin & Z. Y. Liu. 4 females, 1 mj, locality 5, 26-II-2003; 1 female, locality 1, 14-I-2004; 10 females, 6 mj, locality 2, 27-I-2004, coll. Y. Xiong. 1 mj, locality 11, 22-III-2017, coll. Y. Bu.

####### Distribution.

Widely distributed in China (Hainan, Zhejiang, Shanghai, Jiangxi, Anhui, Hubei, Sichuan, Chongqing, Guizhou, Yunnan, Ningxia, Gansu, Shaanxi, Henan, Hebei, Liaoning, Neimenggu).

###### Kenyentulus ciliciocalyci

Taxon classificationAnimaliaProturaBerberentulidae

Yin, 1987

DAEA8EC0-B0B8-599B-8A1F-430EEA90D8D0

####### Material examined.

5 females, locality 1, 27-XI-1984, coll. G. T. Jin & Z. Y. Liu. 1 female, VI-1993; 1 female, IV-1994, locality 1, coll. C. H. Liao. 9 females, 2 males, locality 5, 26-II-2003; 1 female, locality 6, 2-III-2003; 1 female, locality 5, 15-VI-2003; 6 females, 1 male, locality 1, 6-X-2003; 9 females, 2 males, 13 mj, locality 1, 14-I-2004; 7 females, 3 males, locality 1, 15-I-2004; 1 female, locality 1, 14-IV-2004; 2 females, 5 males, 1 mj, locality 1, 15-VII-2004, coll. Y. Xiong. 2 females, 1 male, 2 mj, locality 9, 20-III-2011, coll. Y. Bu & C. W. Huang. 14 females, 10 male, 1 mj, locality 11, 22-III-2017; 3 males, locality 12, 17-X-2017, coll. Y. Bu.

####### Distribution.

Widely distributed in China (Hainan, Zhejiang, Hunan, Sichuan, Chongqing, Guizhou, Yunnan, Shaanxi).

###### Kenyentulus dolichadeni

Taxon classificationAnimaliaProturaBerberentulidae

Yin, 1987

1949F23F-73CC-5029-9781-479910DE8677

####### Material examined.

3 females, locality 2, 14-XI-1984, coll. G. T. Jin & Z. Y. Liu.

####### Distribution.

China (Hainan, Zhejiang, Guangxi, Guizhou, Hubei, Sichuan, Jiangxi).

###### Kenyentulus hainanensis

Taxon classificationAnimaliaProturaBerberentulidae

Yin, 1987

069666C6-53E9-5ED5-ABA9-CF36A40539A9

####### Material examined.

4 females, 1 male, locality 1, 30-XI-1984; 2 females, 2 males, locality 2, 14-XI-1984; 1 male, 2 mj, locality 3, 19-I-1985, coll. G. T. Jin & Z. Y. Liu.

####### Distribution.

China (Hainan, Guangdong).

###### Kenyentulus henanensis

Taxon classificationAnimaliaProturaBerberentulidae

Yin, 1983

C409945E-EC68-5719-9684-C7D26DCF0CB2

####### Material examined.

2 female, 1 male, locality 2, 19-XI-1984, coll. G. T. Jin & Z. Y. Liu.

####### Distribution.

Widely distributed in China (Hainan, Zhejiang, Jiangxi, Henan, Hubei, Guizhou, Yunnan, Ningxia).

###### Kenyentulus japonicus

Taxon classificationAnimaliaProturaBerberentulidae

(Imadate, 1961)

15C9072A-885C-5DDE-9165-525C29C3FC2A

####### Material examined.

2 females, locality 2, 14-XI-1984; 5 females, 5 males, locality 1, 25-XI-1984, coll. G. T. Jin & Z. Y. Liu.

####### Distribution.

Widely distributed in China (Hainan, Zhejiang, Jiangsu, Shanghai, Jiangxi, Anhui, Hunan, Sichuan, Guizhou, Yunnan, Shaanxi); Japan.

###### Kenyentulus jianfengensis

Taxon classificationAnimaliaProturaBerberentulidae

Yin, 1987

2283ED13-750D-521E-9E58-6C9A6E8C237A

####### Material examined.

4 females, locality 1, 1-XII-1984, coll. G. T. Jin & Z. Y. Liu. 2 females, 1 male, 1 mj, locality 1, 6-X-2003; 7 females, 5 males, 2 mj, locality 1, 15-I-2004; 1 mj, locality 3, 27-I-2004; 3 females, 1 males, 1 mj, locality 1, 14-IV-2004; 7 females, 9 males, locality 1, 15-VII-2004, coll. Y. Xiong.

####### Distribution.

China (Hainan, Guizhou).

###### Kenyentulus jinghongensis

Taxon classificationAnimaliaProturaBerberentulidae

Yin, 1983

4C410C8D-934C-58F0-B6BC-C74724136BA0

####### Material examined.

3 females, locality 1, 25-XI-1984, coll. G. T. Jin & Z. Y. Liu.

####### Distribution.

China (Hainan, Yunnan, Guizhou).

###### Kenyentulus minys

Taxon classificationAnimaliaProturaBerberentulidae

Yin, 1983

FFAB7526-BB9F-58A9-828D-930BB2FA5E98

####### Material examined.

2 females, 2 males, locality 1, 19-XI-1984, coll. G. T. Jin & Z. Y. Liu.

####### Distribution.

China (Hainan, Yunnan, Guangxi, Jiangxi)

###### Amphientulus sinensis

Taxon classificationAnimaliaProturaBerberentulidae

Xiong, Xie & Yin, 2005

674EC540-2878-571A-9BF8-006304F5895B

####### Material examined.

1 female, locality 1, 17-XII-2002; 1 female, locality 2; 8 females, 4 males, 1 mj, locality 3, 27-I-2004, coll. Y. Xiong.

####### Distribution.

China (Hainan, Guangdong).

##### Family Sinentomidae Yin, 1965

###### Sinentomon erythranum

Taxon classificationAnimaliaProturaBerberentulidae

Yin, 1965

C38F3FC0-C2C3-5D67-BA34-02520599B07F

####### Material examined.

1 female, 1male, 1 mj, locality 5, 26-II-2003; 1 mj, locality 1, 6-X-2003; 7 females, locality 1, 15-VII-2004, coll. Y. Xiong. 1 female, 1 LI, locality 13, 16-X-2017; 1 LI, locality 12, 17-X-2017, coll. Y. Bu.

####### Distribution.

Widely distributed in South China (Hainan, Shanghai, Jiangsu, Zhejiang, Anhui, Fujian, Guangxi, Guangdong, Hunan, Guizhou, Yunnan).

##### Fujientomidae Yin, 1996

###### Fujientomon dicestum

Taxon classificationAnimaliaProturaFujientomidae

Yin, 1977

882EFFBD-5783-527D-ABCB-25EEC2A623E2

####### Material examined.

1 female, locality 5, 26-II-2003, coll. Y. Xiong.

####### Distribution.

China (Hainan, Shanghai, Jiangsu, Zhejiang, Anhui, Ningxia).

##### Eosentomidae Berlese, 1909

###### Eosentomon actitum

Taxon classificationAnimaliaProturaEosentomidae

Zhang, 1983

8A312847-ED66-526E-8B96-A2742CA7D017

####### Material examined.

8 females, 6 males, 2 mj, locality 1, XII-1984; 24 females, 24 males, 27 mj, locality 3, 23-I-1985, coll. G. T. Jin & Z. Y. Liu. 2 females, 3 males, locality 8, 22-II-2003, coll. Y. Xiong. 2 females, 3 mj, locality 11, 22-III-2017, coll. Y. Bu.

####### Distribution.

China (Hainan, Guangdong, Sichuan).

###### Eosentomon hainanense

Taxon classificationAnimaliaProturaEosentomidae

Yin, 1986

BF9B93BA-485A-5E00-B4F6-27E47728AEE5

####### Material examined.

40 females, 33 males, 2 mj, locality 1, 25-XI-1984, coll. G. T. Jin & Z. Y. Liu. 2 females, VI-1993, locality 1, coll. C. H. Liao. 1 female, 5 males, locality 8, 22-II-2003; 4 females, 1 male, locality 6, 2-III-2003; 1 female, 3 males, 4 mj, locality 5, 15-VI-2003; 19 female, 15 males, 8 mj, locality 1, 7-X-2003; 33 females, 31 males, 15 mj, locality 1, 14-I-2004; 1 male, locality 3, 960 m elev., 27-I-2004; 8 females, 8 males, 1 mj, locality 1, 15-VII-2004, coll. Y. Xiong.

####### Distribution.

China (Hainan, Yunnan).

###### Eosentomon iban

Taxon classificationAnimaliaProturaEosentomidae

Imadate, 1965

CDE43EFB-E528-58D3-A558-6A8D8C89DCAE

####### Material examined.

2 females, 2 males, locality 1, 27-XI-1984, coll. G. T. Jin & Z. Y. Liu.

####### Distribution.

China (Hainan); Malaysia, Brunei.

###### Eosentomon jinhongense

Taxon classificationAnimaliaProturaEosentomidae

Yin, 1982

E06C531A-0D94-5E98-BFA5-7851EBFB78FD

####### Material examined.

2 females, 1 male, locality 2, 14-XI-1984, coll. G. T. Jin & Z. Y. Liu.

####### Distribution.

China (Hainan, Yunnan).

###### Eosentomon margarops

Taxon classificationAnimaliaProturaEosentomidae

Yin & Zhang, 1982

E6A367D6-24B0-5D55-A550-8D1BE9B2EDB6

####### Material examined.

1 female, 2 males, 1 mj, locality 5, 26-II-2003; 1 female, 1 male, 2 mj, locality 5, 15-VI-2003; 1 female, 2 males, locality 1, 6-X-2003; 6 females, 3 males, 1 mj, locality 1, 15-I-2004; 2 females, 1 male, 1 mj, locality 2, 820 m elev., 27-I-2004; 3 females, 2 males, 2 mj, locality 3, 1000 m elev., 27-I-2004; 2 females, 1 male, locality 1, 14-IV-2004; 2 females, locality 1, 15-VII-2004, coll. Y. Xiong. 1 female, locality 10, 23-III-2017, coll. Y. Bu.

####### Distribution.

China (Hainan, Guangdong, Sichuan).

###### Eosentomon novemchaetum

Taxon classificationAnimaliaProturaEosentomidae

Yin, 1965

8AE595C2-7549-5F4F-A1E8-A80214AFDCD8

####### Material examined.

1 female, locality 11, 22-III-2017, coll. Y. Bu.

####### Distribution.

China (Hainan, Shanghai, Jiangsu, Anhui, Jiangxi, Liaoning, Shaanxi)

###### Eosentomon orientale

Taxon classificationAnimaliaProturaEosentomidae

Yin, 1965

1694DE9C-605E-5ACD-B2F5-C520818FF429

####### Material examined.

1 female, locality 1, 25-XI-1984, coll. G. T. Jin & Z. Y. Liu.

####### Distribution.

Widely distributed in China (Hainan, Shanghai, Jiangsu, Zhejiang, Anhui, Jiangxi, Hubei, Hunan, Guangxi, Guangdong, Sichuan, Chongqing, Guizhou, Ningxia, Shaanxi, Liaoning).

###### Eosentomon sakura

Taxon classificationAnimaliaProturaEosentomidae

Imadate & Yosii, 1959

A9CD6AEA-1164-5FF4-BB40-7D12DF2E5C98

####### Material examined.

5 females, 3 males, locality 1, 25-XI-1984, coll. G. T. Jin & Z. Y. Liu. 13 females, 7 males, 9 mj, locality 8, 22-II-2003; 1 female, 1 mj, locality 5, 20-I-2003; 8 females, 13 males, 7 mj, locality 5, 26-II-2003; 1 female, 1 male, locality 6, 2-III-2003; 2 females, 5 males, 2 mj, locality 5, 15-VI-2003; 2 females, 1 mj, locality 1, 6-X-2003; 3 females, 1 male, 2 mj, locality 7, 13-VII-2003; 5 females, 2 males, 2 mj, locality 1, 15-I-2004; 46 females, 45 males, 29 mj, locality 4, 23-I-2004; 12 females, 20 males, 10 mj, locality 2, 820 m elev., 27-I-2004; 5 females, 5 males, 2 mj, locality 1, 500 m elev., 27-I-2004, coll. Y. Xiong. 1 male, locality 2, 20-III-2011, coll. Y. Bu & C. W. Huang. 8 females, 7 males, 3 mj, locality 11, 22-III-2017, coll. Y. Bu.

####### Distribution.

Widely distributed in China (Hainan, Shanghai, Jiangsu, Zhejiang, Anhui, Jiangxi, Hubei, Hunan, Guangxi, Guangdong, Yunnan, Sichuan, Fujian, Guizhou, Taiwan, Hong Kong, Shaanxi).

###### Eosentomon shanum

Taxon classificationAnimaliaProturaEosentomidae

(Zhang, 1984)

1D0B51EB-5F67-5C3B-A074-D3F2CB089F16

####### Material examined.

2 females, 1 male, locality 4, 23-I-2004; 1 female, 2 males, locality 1, 14-IV-2004; 5 females, 1 mj, locality 1, 15-VII-2004, coll. Y. Xiong.

####### Distribution.

China (Hainan, Guangxi, Hunan, Jiangxi).

###### Eosentomon spanum

Taxon classificationAnimaliaProturaEosentomidae

Yin, 1986

508839A1-4D50-5849-9BE9-1818C48F2BDC

####### Material examined.

1 female, 1 male, locality 1, 27-XI-1984, coll. G. T. Jin & Z. Y. Liu.

####### Distribution.

China (Hainan).

###### Eosentomon tropicum

Taxon classificationAnimaliaProturaEosentomidae

Yin, 1986

8DE2605A-639E-515E-882F-4E8A98B669C5

####### Material examined.

5 females, locality 1, 25-XI-1984, coll. G. T. Jin & Z. Y. Liu. 1 female, VII-1993, locality 1, coll. C. H. Liao. 1 female, 3 males, 3 mj, locality 8, 22-II-2003; 4 females, 3 males, 4 mj, locality 1, 6-X-2003; 2 mj, locality 1, 15-I-2004; 3 females, 1 male, locality 1, 15-VII-2004, coll. Y. Xiong. 2 females, locality 11, 22-III-2017, coll. Y. Bu.

####### Distribution.

China (Hainan).

###### Eosentomon xishaense

Taxon classificationAnimaliaProturaEosentomidae

Yin, 1988

8D963A9F-C583-5F19-9E45-7569CB835357

####### Material examined.

2 females, locality 1, 27-XI-1984, coll. G. T. Jin & Z. Y. Liu. 1 mj, VI-1993, locality 1, coll. C. H. Liao. 1 female, locality 5, 20-I-2003; 3 mj, locality 1, 14-IV-2004; 4 females, 2 males, locality 1, 15-VII-2004, coll. Y. Xiong. 1 female, locality 9, 20-III-2011, coll. Y. Bu & C. W. Huang. 2 females, 1 male, 1 mj, locality 11, 22-III-2017; 1 male, locality 10, 23-III-2017, coll. Y. Bu.

####### Distribution.

China (Hainan, Xisha Islands, Yongxing Island).

###### Eosentomon yanshanense

Taxon classificationAnimaliaProturaEosentomidae

Yin & Zhang, 1982

AFFD70A7-FBA1-5D48-8985-47CBCD7068C6

####### Material examined.

2 females, locality 1, 25-XI-1984, coll. G. T. Jin & Z. Y. Liu. 2 females, 4 males, 7 mj, locality 6, 2-III-2003; 9 females, 3 males, 10 mj, locality 4, 23-I-2004; 2 females, 2 males, locality 1, 14-IV-2004; 2 females, 1 mj, locality 1, 15-VII-2004, coll. Y. Xiong.

####### Distribution.

China (Hainan, Guangxi, Guangdong, Fujian, Jiangxi, Hunan, Hubei, Yunnan).

###### Eosentomon zhanjiangense

Taxon classificationAnimaliaProturaEosentomidae

Zhang, 1983

C3271BDF-683F-53FD-A4FF-13D10DECCC7B

####### Material examined.

2 females, locality 1, 19-I-1985, coll. G. T. Jin & Z. Y. Liu. 1 females, 2 males, locality 1, 14-IV-2004, coll. Y. Xiong.

####### Distribution.

China (Hainan, Guangdong).

###### Anisentomon hainanense

Taxon classificationAnimaliaProturaEosentomidae

Xiong, Bu & Yin, 2008

157D9716-E249-5B6E-B3D3-F4891A8D3716

####### Material examined.

1 female, 1 male, locality 1, 6-X-2003, coll. Y. Xiong.

####### Distribution.

China (Hainan).

###### Anisentomon quadrisetum

Taxon classificationAnimaliaProturaEosentomidae

Zhang & Yin, 1981

F3177C7D-A610-5DC4-AC41-62669FE5DE1D

####### Material examined.

1 female, 1 male, locality 1, 7-X-2003, 14 females; 7 males, locality 1, 14-I-2004, coll. Y. Xiong. 1 male, 1 mj, locality 11, 22-III-2017, coll. Y. Bu.

####### Distribution.

China (Hainan, Guangxi, Guangdong).

###### Neanisentomon yuenicum

Taxon classificationAnimaliaProturaEosentomidae

Zhang & Yin, 1984

74612738-A6E7-56F1-9E5E-B5C92570CAB1

####### Material examined.

1 female, locality 1, 14-IV-2004, coll. Y. Xiong. 1 female, 1 male, locality 10, 23-III-2017, coll. Y. Bu.

####### Distribution.

China (Hainan, Guangdong).

###### Paranisentomon tuxeni

Taxon classificationAnimaliaProturaEosentomidae

(Imadate & Yosii, 1959)

06343631-9405-5DCF-B99B-AE12D3DDF16B

####### Material examined.

6 females, 1 mj, locality 3, 1000 m elev., 27-I-2004, coll. Y. Xiong.

####### Distribution.

China (Hainan, Hubei, Hunan, Jiangxi, Anhui, Guizhou, Shaanxi).

###### Pseudanisentomon paurophthalmum

Taxon classificationAnimaliaProturaEosentomidae

Zhang & Yin, 1984

CF1EE964-6F9F-5B23-BBCA-62A1A810F6CA

####### Material examined.

1 female, 1 mj, locality 11, 22-III-2017, coll. Y. Bu.

####### Distribution.

China (Hainan, Guangxi).

###### Pseudanisentomon molykos

Taxon classificationAnimaliaProturaEosentomidae

Zhang & Yin, 1984

8037AC5C-72A5-578C-89B5-5D666247C7AB

####### Material examined.

5 females, 2 males, 1 mj, locality 1, 6-X-2003; 1 female, locality 1, 14-IV-2004, coll. Y. Xiong.

####### Distribution.

China (Hainan, Guangdong, Guangxi, Yunnan).

###### Pseudanisentomon sininotiale

Taxon classificationAnimaliaProturaEosentomidae

Zhang & Yin, 1984

3022B716-9730-512B-8167-F277C02FE66C

####### Material examined.

2 females, locality 1, 27-XI-1984, coll. G. T. Jin & Z. Y. Liu. 2 females, 4 males, locality 4, 23-I-2004; 1 male, locality 3, 600 m elev., 27-I-2004, coll. Y. Xiong.

####### Distribution.

China (Hainan, Guangxi, Hunan).

####### Discussion.

The 34 species of Protura recorded from Hainan Island belong to 11 genera and five families (Protentomidae, Berberentulidae, Sinentomidae, Fujientomidae and Eosentomidae) (Table 5). Most species (91%) belong to Eosentomidae (21 species) and Berberentulidae (10 species), while the other three families are represented by one species each. Both Berberentulidae and Eosentomidae are widely distributed in China and have high species richness. In contrast, Sinentomidae, Protentomidae, and Fujientomidae each has fewer species occurring in China. Undoubtedly, proturans found in Hainan Island are mainly related to the fauna of Oriental Region and are distinctly different from those from Russian Far East and Siberia (Bu et al. 2014; Shrubovych 2014), which are dominated by the family Acerentomidae.

**Table 5.** The list of proturan species from Hainan Island and their distribution in Hainan Island and three neighboring mainland provinces.

**Classification**	**Species**	**Hainan**	**Guangdong**	**Guangxi**	**Yunnan**
**Acerentomata Yin, 1996**					
**Protentomidae Ewing, 1936**					
*Paracondeellum* Yin, Xie & Zhang, 1994	*P. paradisum* sp. n.*	11**			
**Berberentulidae Yin, 1983**					
*Baculentulus* Tuxen,1977	*B. tienmushanensis* (Yin, 1963)	2, 3, 5, 11			+
*Kenyentulus* Tuxen, 1981	*K. ciliciocalyci* Yin, 1987	1, 5, 6, 9, 11, 12			+
	*K. dolichadeni* Yin, 1987	2			
	*K. hainanensis* Yin, 1987	1, 3	+		
	*K. henanensis* Yin, 1983	2			+
	*K. japonicus* (Imadate, 1961)	2			+
	*K. jianfengensis* Yin, 1987	1, 2			
	*K. jinghongensis* Yin, 1983	1			+
	*K. minys* Yin, 1983	1		+	+
*Amphientulus* Tuxen, 1981	*A. sinensis* Xiong, Xie & Yin, 2005	1, 3	+		
**Sinentomata Yin, 1996**					
**Sinentomidae Yin, 1965**					
*Sinentomon* Yin, 1965	*S. erythranum* Yin, 1965	1, 5, 12, 13	+	+	+
**Fujientomidae Yin, 1996**					
*Fujientomon* Yin, 1977	*F. dicestum* Yin, 1977	5			
**Eosentomata Yin, 1996**					
**Eosentomidae Berlese, 1909**					
*Eosentomon* Berlese, 1909	*E. actitum* Zhang, 1983	1, 3, 8, 11	+		
	*E. hainanense* Yin, 1986*	1, 3, 5, 6, 8	+		+
	*E. iban* Imadate, 1965	1			
	*E. jinhongense* Yin, 1982	2			+
	*E. margarops* Yin & Zhang, 1982	1, 2, 3, 5, 10	+		
	*E. novemchaetum* Yin, 1965	11			
	*E. orientale* Yin, 1965	1	+	+	
	*E. sakura* Imadate & Yosii, 1959	1, 2, 4–8, 11	+	+	+
	*E. shanum* (Zhang, 1984)	1, 4		+	
	*E. spanum* Yin, 1986*	1			
	*E. tropicum* Yin, 1986*	1, 8, 11			
	*E. xishaense* Yin, 1988	1, 5, 9–11			
	*E. yanshanense* Yin & Zhang, 1982	1, 4, 6	+	+	+
	*E. zhanjiangense* Zhang, 1983	1	+		
*Anisentomon* Yin, 1977	*A. hainanense* Xiong, Bu & Yin, 2008*	1			
	*A. quadrisetum* Zhang & Yin, 1981	1, 11	+	+	
*Neanisentomon* Zhang & Yin, 1984	*N. yuenicum* Zhang & Yin, 1984	1, 10	+		
*Paranisentomon* Zhang & Yin, 1984	*P. tuxeni* (Imadate & Yosii, 1959)	3			
*Pseudanisentomon* Zhang & Yin, 1984	*P. paurophthalmum* Zhang & Yin, 1984	11		+	
	*P. molykos* Zhang & Yin, 1984	1	+	+	
	*P. sininotiale* Zhang & Yin, 1984	1, 4		+	

* Species known only from Hainan Island so far. ** Numbers indicate the localities given in Table 1.

By comparing the species distribution, we found that the Protura fauna of Hainan Island is closely related to those of neighboring mainland regions (Yin 1999; Szeptycki 2007), and there are 13, 10, and 11 species shared with Guangdong, Guangxi, and Yunnan provinces, respectively (Fig. 1; Table 5), which is consistent with the geological history of Hainan Island (Wang 1991; Zhang and Fang 2012). Until the Quaternary period (2.5 million years ago), Hainan Island was still connected with Leizhou Peninsula of Guangdong Province. In the Middle Pleistocene, fault depression led to the separation of Hainan Island from the mainland. With sea level fluctuations, Hainan Island was connected to or separated from the mainland for several times. Since the end of the Quaternary period, due to the drastically rise of sea level, Hainan Island has been separated from the mainland without interruption.

Among the mainland regions neighboring Hainan Island, the Protura fauna of Yunnan Province has been systematically studied (Zhang et al. 1996; Yin et al. 2000), and nearly 80 species were reported from that province, with the Berberentulidae and Ensentomidae having fairly high diversity (Zhang et al. 1996; Yin et al. 2000). In this study, we found the diversity of Protura fauna from Hainan Island is very similar to that from Yunnan Province. The only difference is the presence of family Hesperentomidae in Yunnan, which is absent in Hainan Island.

Sampling localities in Hainan Island are still sparse, and additional collection of proturans should be made in the future, so as to reveal the true diversity and provide a better understanding of the biogeography of Protura on the Hainan Island.

## Supplementary Material

XML Treatment for Paracondeellum

XML Treatment for Paracondeellum paradisum

XML Treatment for Paracondeellum dukouense

XML Treatment for Paracondeellum paradisum

XML Treatment for Baculentulus tienmushanensis

XML Treatment for Kenyentulus ciliciocalyci

XML Treatment for Kenyentulus dolichadeni

XML Treatment for Kenyentulus hainanensis

XML Treatment for Kenyentulus henanensis

XML Treatment for Kenyentulus japonicus

XML Treatment for Kenyentulus jianfengensis

XML Treatment for Kenyentulus jinghongensis

XML Treatment for Kenyentulus minys

XML Treatment for Amphientulus sinensis

XML Treatment for Sinentomon erythranum

XML Treatment for Fujientomon dicestum

XML Treatment for Eosentomon actitum

XML Treatment for Eosentomon hainanense

XML Treatment for Eosentomon iban

XML Treatment for Eosentomon jinhongense

XML Treatment for Eosentomon margarops

XML Treatment for Eosentomon novemchaetum

XML Treatment for Eosentomon orientale

XML Treatment for Eosentomon sakura

XML Treatment for Eosentomon shanum

XML Treatment for Eosentomon spanum

XML Treatment for Eosentomon tropicum

XML Treatment for Eosentomon xishaense

XML Treatment for Eosentomon yanshanense

XML Treatment for Eosentomon zhanjiangense

XML Treatment for Anisentomon hainanense

XML Treatment for Anisentomon quadrisetum

XML Treatment for Neanisentomon yuenicum

XML Treatment for Paranisentomon tuxeni

XML Treatment for Pseudanisentomon paurophthalmum

XML Treatment for Pseudanisentomon molykos

XML Treatment for Pseudanisentomon sininotiale

## References

[B1] Bu Y Gao Y Luan YX Yin WY (2012) Progress on the systematic study of basal Hexapoda. Chinese Bulletin of Life Sciences 24(2): 130–138. [in Chinese with English abstract]

[B2] Bu Y Potapov MB Yin WY (2014) Systematic and biogeographical study of Protura (Hexapoda) in Russian Far East: new data on high endemism of the group. ZooKeys 424: 19–57. 10.3897/zookeys.424.7388 PMC4106100 25061395

[B3] Bu Y Qian CY Luan YX (2017) Three newly recorded species of Acerentomata (Hexapoda: Protura) from China, with analysis of DNA barcodes. Entomotaxonomia 39(1): 1–14. 10.11680/entomotax.2017001

[B4] Bu Y Yin WY (2007) Two new species of *Hesperentomon* Price, 1960 from Qinghai Province, northwestern China (Protura: Hesperentomidae). Acta Zootaxonomica Sinica 32(3): 508–514.

[B5] Galli L Shrubovych J Bu Y Zinni M (2018) Genera of the Protura of the world: diagnosis, distribution, and key. ZooKeys 772: 1–45. 10.3897/zookeys.772.24410 PMC6045683 30018507

[B6] Huang FS (2002) Insect Fauna of Hainan Forest. Science Press, Beijing, 1063 pp. [In Chinese]

[B7] Qian CY Bu Y Luan YX (2018) DNA barcoding and an updated key to the genus *Hesperentomon* (Protura: Acerentomata: Hesperentomidae), with a new species from Northwest China. Zootaxa 4462(4): 523–534. 10.11646/zootaxa.4462.4.5 30313457

[B8] Rusek J Shrubovych J Szeptycki A (2012) Head porotaxy and chaetotaxy of order Acerentomata (Protura). Zootaxa 3262: 54–61. 10.11646/zootaxa.3262.1.5

[B9] Shrubovych J (2014) Identification and character analysis of the Acerentomidae (Protura) of the northeastern Palearctic (Protura: Acerentomidae). Zootaxa 3755(2): 136–164. 10.11646/zootaxa.3755.2.2 24869813

[B10] Szeptycki A (2007) Catalogue of the world Protura. Acta Zoologica Cracoviensia 50(1): 1–210. 10.3409/000000007783995417

[B11] Tang B Yin WY (1988) Three new species of Protura from Sichuan Province. Zoological Research 9(3): 309–315. [in Chinese with English abstract]

[B12] Wang XF (1991) Geology of Hainan Island III, Structural Geology. Science Press, Beijing, China, 138 pp. [in Chinese]

[B13] Xiong Y (2005) The community diversity of soil animals in the tropical and subtropical forests and the phylogeny of Collembola. PhD Thesis, East China Normal University, Shanghai, 139 pp. [in Chinese with English abstract]

[B14] Xiong Y Bu Y Yin WY (2008) A new species of *Anisentomon* from Hainan, Southern China (Protura: Eosentomidae). Zootaxa 1727: 39–43. 10.11646/zootaxa.1727.1.4

[B15] Xiong Y Xie RD Yin WY (2005) First record of the genus *Amphientulus* Tuxen, 1981 (Protura: Acerentomidae) from China, with description of a new species. The Raffles Bulletin of Zoology 53(1): 1–5.

[B16] Yin WY (1986) Three new species and a new record of *Eosentomon* from Hainan Island, China (Protura: Eosentomidae). Contribution from Shanghai Institute of Entomology 6: 135–140. [in Chinese with English abstract]

[B17] Yin WY (1987) Four new species of *Kenyentulus* from hainan Island. Zoological Research 8(2): 149–157. [in Chinese with English abstract]

[B18] Yin WY (1999) Fauna Sinica. Arthropoda. Protura. Science Press, Beijing, 510 pp.

[B19] Yin WY (2002) Protura. In: Huang FS (Ed.) Insect Fauna of Hainan Forest.Science Press, Beijing, 24–27. [In Chinese]

[B20] Yin WY Xie R Imadaté G (2000) Protura of Yunnan, Southwest China, with description of four new species (Protura: Eosentomata), In: Aoki J, Yin WY, Imadaté G (Eds) Taxonomical Studies on the Soil Fauna of Yunnan Province in Southwest China, Tokai University Press, Tokyo, 117–131.

[B21] Yin WY Xie R Zhang J (1994) Phylogeny and biogeography of *Condeellum* group. (Protura: Protentomidae). Entomologia Sinica 1(3): 195–240. 10.1111/j.1744-7917.1994.tb00245.x

[B22] Yin ZW Li LZ Wu C (2015) New and little known species of *Zorotypus* Silvestri (Zoraptera: Zorotypidae) from China. Zootaxa 4007(4): 557–566. 10.11646/zootaxa.4007.4.6 26623832

[B23] Zhang J Xie R Yin WY (1996) Study on diversity of Protura from Yunnan province. Zoological Research 17(2): 139–146. [in Chinese with English abstract]

[B24] Zhang LS Fang XQ (2012) Paleogeography of China, the Formation of Natural Environment in China. Science Press, Beijing, 425 pp. [In Chinese]

